# Wearable and Implantable Electroceuticals for Therapeutic Electrostimulations

**DOI:** 10.1002/advs.202004023

**Published:** 2021-02-19

**Authors:** Yin Long, Jun Li, Fan Yang, Jingyu Wang, Xudong Wang

**Affiliations:** ^1^ Department of Material Science and Engineering University of Wisconsin–Madison Madison WI 53706 USA

**Keywords:** electroceuticals, nanogenerators, self‐powered bioelectronics, therapeutic electrostimulations, wearable and implantable

## Abstract

Wearable and implantable electroceuticals (WIEs) for therapeutic electrostimulation (ES) have become indispensable medical devices in modern healthcare. In addition to functionality, device miniaturization, conformability, biocompatibility, and/or biodegradability are the main engineering targets for the development and clinical translation of WIEs. Recent innovations are mainly focused on wearable/implantable power sources, advanced conformable electrodes, and efficient ES on targeted organs and tissues. Herein, nanogenerators as a hotspot wearable/implantable energy‐harvesting technique suitable for powering WIEs are reviewed. Then, electrodes for comfortable attachment and efficient delivery of electrical signals to targeted tissue/organ are introduced and compared. A few promising application directions of ES are discussed, including heart stimulation, nerve modulation, skin regeneration, muscle activation, and assistance to other therapeutic modalities.

## Graphical abstract

An overview of the most recent innovations in wearable and implantable electroceuticals (WIEs) with focus on nanogenerator (NG) power sources, advanced conformable electrodes, and efficient electrostimulation on targeted organs and tissues is presented. The NG‐based technology is foreseeable to transform the concurrent WIEs toward the next generation of precision electrotherapy in the near future.

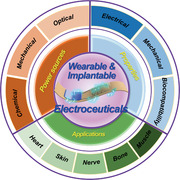

## 1 Introduction

Existence of free ions, dipoles, and polarized molecules inside human body enables cells tissues and organs to generate and conduct electricity. The small bioelectricity plays a vital role of regulating physiological processes and sustaining human life. Discovery of the interplay between electricity and biology could date back to the ancient Rome when people found that the torpedo fish (electric ray) could be applied to relieve chronic pains of body. The direct observation of the twitching responses from a dead frog's leg under electrical impulses by biologist Luigi Aloisio Galvani led to the awareness of the electricity influences to biological systems. This groundbreaking discovery also sparked a new field of using electrostimulation (ES) devices (electroceuticals) to study electrophysiology, treat diseases, and achieve many therapeutic purposes. Till now, electroceuticals have attracted intensive attentions from both academia and industry due to their indispensable application potential for improving the quality of life and prolonging lifespan.^[^ ^1^, ^2^, ^3^ ^]^ For instance, external ES could accelerate chronic wound healing, improve rehabilitation after spinal cord injury, and treat muscle spasms and pains. These ES devices have also contributed significantly to our knowledge of the physiological processes in the human body, including the complicated mechanisms of neural communication, memory, and control. In turn, they provided new solutions to the treatments of complications that could be hardly cured by other means.^[^ ^1^ ^]^

Compared to conventional clinical electroceutical devices, the state‐of‐the‐art wearable and implantable electroceutical (WIE) devices are implementing new design principles, new flexible materials, and innovative fabrication techniques to achieve ultimate body compatibility and comfortableness with superb performance. In contrast to the rapidly evolved microelectronic technologies, the developments of suitable flexible/biocompatible power supplies for WEIs are relatively slow. Till recent years, flexible and light‐weight batteries, supercapacitors, and energy harvesters started to emerge with acceptable energy density and power output, allowing for reaching desired flexibility and size miniaturization of WIEs. Particularly, nanogenerators (NGs) as a new technology for converting biomechanical energy into electricity could function as the sole power source WIEs or provide complementary power to other power source (such as batteries) to prolong the operational lifespan. New design principles of WIEs are in coordination with physiological and pathological properties of relevant tissue. Specifically, mechanical properties such as flexibility and stretchability are required to match/adapt to that of soft tissues in order to minimize the risk of tissue injury and reduce burdens on irrelevant organ functions. In addition, electrical properties vital for efficient stimulation of excitable tissues are adjusted to allow best performance. The transferred charges from the electrode to the cell need to be strong enough to depolarize its membrane above a certain threshold level but avoided Faradic conduction, which will cause electrode corrosion and generate toxic species.^[^ ^2^, ^3^ ^]^ Moreover, long‐term biocompatibility and biosafety are also crucial for translation of the WIEs to real clinical applications.

**Figure 1:**
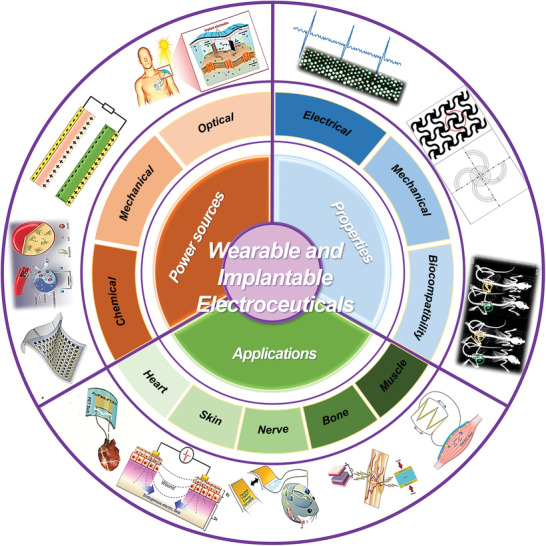
Overview of design and innovation of state‐of‐the‐art WIEs based on power sources, properties, and specific applications. Examples of WIE power sources include the three main mechanisms via chemical energy (e.g., battery, BFC), mechanical energy (e.g., NG), and optical energy (e.g., photovoltaics) conversion. The main fundamental properties of WIEs to be improved include mechanical, electrical, and biological properties to achieve desired compatibility at the biointerfaces, and long‐term reliable and stable operation. Representative application directions of WIEs include cardiovascular system, brain‐nerve system, skin regeneration, bone healing, and ES‐assisted muscle recovery. Reproduced with permission.^[^ ^41^ ^]^ Copyright 2017, Elsevier. Reproduced with permission.^[^ ^42^ ^]^ Copyright 2019, Wiley‐VCH. Reproduced with permission.^[^ ^12^ ^]^ Copyright 2013, Springer Nature. Reproduced with permission.^[^ ^59^ ^]^ Copyright 2020, Wiley‐VCH. Reproduced with permission.^[^ ^49^ ^]^ Copyright 2014, Wiley‐VCH. Reproduced with permission.^[^ ^190^ ^]^ Copyright 2018, American Chemical Society. Reproduced with permission.^[^ ^183^ ^]^ Copyright 2015, Royal Society of Chemistry. Reproduced with permission.^[^ ^213^ ^]^ Copyright 2019, Taylor & Francis Group. Reproduced with permission.^[^ ^139^ ^]^ Copyright 2019, Wiley‐VCH.

In general, a WIE typically consists of a power source providing electric stimulations and sets of electrodes to deliver electric stimulations to targeted cells or tissues to achieve desired therapeutic purposes. WIEs are experiencing fast advances, and their potential implementations are extending to areas such as brain‐nerve systems, cardiovascular systems, skin regeneration, bone healing, and many other ES‐assisted diseases. While wearable and implantable medical devices (WIMDs) are broadly reviewed in current literature,^[^ ^4^, ^5^, ^6^, ^7^, ^8^, ^9^ ^]^ most of them focused on the progress of WIMDs from perspectives of healthcare monitoring/neural recording,^[^ ^6^ ^]^ flexible and stretchable device engineering,^[^ ^5^ ^]^ or specific types of medical functions (e.g., photonics,^[^ ^4^ ^]^ biodegradable/bioresorbable systems,^[^ ^7^, ^8^ ^]^ and NGs^[^ ^9^ ^]^). Distinguishably, this review article is dedicated to the state of the art of self‐powered WIEs enabled by NG technology and their unique therapeutic implementations (**Figure** **1**). We first introduce NG as a state‐of‐art power approach of WIEs. Design principles (mechanical, electrical, and biological) to endow the WIEs with biomimetic properties to improve both compatibility and performance at the biointerfaces are then systematically discussed with in vivo demonstrations. Afterwards, different WIEs examples, particularly the self‐powered WIEs, targeted at heart, skin, nerve, and bone are reviewed. Both treatment mechanisms and posttreatment effects/results are analyzed. At the end, challenges, opportunities as well as perspectives existing in this field are summarized and proposed.

## 2 Power Supply and NGs

The power supply is an essential component of WIE devices that still takes the majority of volume and weight of entire system. Primary batteries are recognized as the most reliable power source for WIE devices. However, conventional batteries are usually rigid, bulky, and may contain hazardous constituent materials. The limited lifetime of batteries also brings a potential challenge for replacement if implanted.^[^ ^10^, ^11^ ^]^ Besides, effectively minimizing the foreign body responses (FBRs) poses additional challenges to the development of implanted systems in terms of biological, mechanical, and chemical compatibilities. Nevertheless, batteries are still the most widely used component for powering WIEs. Meanwhile, extensive efforts have been devoted to developing new electrode materials and designing battery architectures to satisfy additional requirements for in vivo applications.^[^ ^12^ ^]^ Compared to batteries, supercapacitors offer an alternative energy storage option for WIEs. They can be more easily designed into flexible, stretchable, and biodegradable systems, due to their relatively simple architecture and electrochemistry.^[^ ^13^, ^14^, ^15^, ^16^ ^]^ Despite many initial successes in proof‐of‐concept demonstration, current wearable/implantable supercapacitors are still at their early stage. Much more research efforts on improving their energy density, maintaining a stable output, raising the working voltage, and sustaining a long‐term biocompatibility should be taken toward the future practical applications.

Wireless in vivo charging is becoming an increasingly popular approach to enable long‐term operation of biomedical devices including WIEs.^[^ ^17^, ^18^, ^19^, ^20^, ^21^, ^22^ ^]^ Electromagnetic energy and ultrasound energy transfer are the two common mechanisms for wireless charging. Electromagnetic charging has the capability of transferring watt‐level power to medical devices.^[^ ^23^ ^]^ However, challenges that limit the in vivo application of this technology are also nontrivial, such as large coils, small transferring distance, complex electronic circuits, high susceptibility to other electromagnetic interference, and high attenuation of electromagnetic wave in tissues.^[^ ^17^, ^24^, ^25^ ^]^ Ultrasound power transferring is effective over a longer distance (up to ≈50cm). Typically, high‐performance piezoelectric materials are used as a receiver to the ultrasonic energy and convert it to electricity.^[^ ^26^ ^]^ This technology is often associated with low energy transferring efficiency, and potential tissue damage due to acoustic impedance mismatch between tissue and transducer.^[^ ^27^, ^28^ ^]^ The material toxicity is also a concern.

Biofuel cells (BFCs) are considered as another promising alternative power source, which are based on the transformation of chemical energy from living organism directly into electricity via redox reaction.^[^ ^29^ ^]^ The enzymatic BFCs have been extensively studied in biological organs and tissues of invertebrates such as snail,^[^ ^30^ ^]^ clam,^[^ ^31^ ^]^ and lobster,^[^ ^32^ ^]^ and vertebrates, such as rats.^[^ ^33^, ^34^ ^]^ In addition to glucose BFC, other common biofuels such as lactate,^[^ ^35^ ^]^ pyruvate, fatty, succinate, and amino acids^[^ ^36^, ^37^, ^38^ ^]^that are harvested from biofluids of sweat, tears, or blood^[^ ^39^, ^40^ ^]^ have been also reported in cells. The merits of wearable or implantable BFCs are obvious, including renewable biocatalysts, abundant, green, safe fuels, and biocompatible. Nevertheless, their very limited lifetime and stability, and low electrical output are large roadblocks toward practical applications.

Different from the abovementioned energy strategies, harvesting energy locally may serve a more compatible and sustainable way for powering WIEs. Among a few accessible energy sources,^[^ ^41^, ^42^ ^]^ biomechanical energy is an abundant and primary source that exists widely in human bodies. NG, an emerging technology based on nanoscale electromechanical coupling, is effective and versatile for directly converting available biomechanical energy in human body into useful electricity. The NG technology has a good potential for long‐term in vivo operation. The longest in vivo operation of NG has been demonstrated for 6 months.^[^ ^43^ ^]^ Ex vivo endurance tests suggested years‐long lifespan, reaching the same level of lifetime of other energy‐storage technologies.^[^ ^44^ ^]^ It, therefore, possesses huge promises as a power supply for WIEs and many more biomedical electronic devices. In this section, our discussion will focus on the up‐to‐date achievements in the development of implantable and degradable NGs, including both piezoelectric nanogenerators (PENGs) and triboelectric nanogenerators (TENGs) as an innovative sustainable power supply strategy for WIEs.

### 2.1 Piezoelectric Nanogenerator

**Figure 2:**
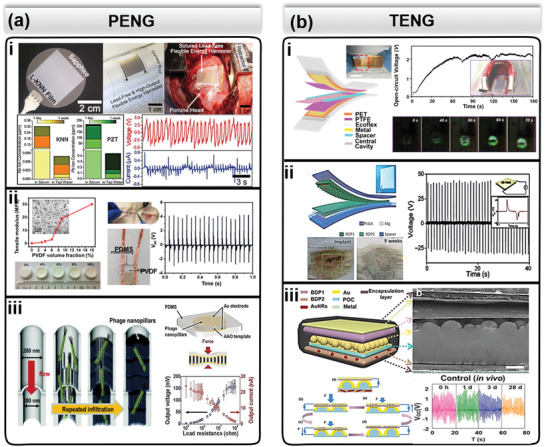
Flexible and implantable NGs. a) Summary of PENG: i) A representative high‐performance, lead‐free, KNN‐based PENG with ultrathin thickness that could be conformally attached to a porcine heart for harvesting cardiac energy. Reproduced with permission.^[^ ^56^ ^]^ Copyright 2017, American Institute of Physics. ii) Mesoporous soft PVDF‐based PENG with tunable mechanical compliance and its in vivo performance implanted under rat skin. Reproduced with permission.^[^ ^57^ ^]^ Copyright 2016, Wiley‐VCH. Reproduced with permission.^[^ ^44^ ^]^ Copyright 2016, Elsevier. iii) PENG assembled by vertically aligned M13 bacteriophage virus with appreciable electricity outputs. Reproduced with permission.^[^ ^61^ ^]^ Copyright 2015, Royal Society of Chemistry. b) Summary of TENG: i) A representative ultrastretchable TENG with tissue‐comparable modulus harvesting rat breathing energy. Reproduced with permission.^[^ ^76^ ^]^ Copyright 2018, American Chemical Society. ii) A typical TENG consisting of synthetic biodegradable materials that could be fully disintegrated inside rat. Reproduced with permission.^[^ ^80^ ^]^ Copyright 2016, American Association for the Advancement of Science. iii) A designated TENG with switchable degradation capability enabled by IR‐responsive gold nanorods embedded in biodegradable polymers matrix. Reproduced with permission.^[^ ^84^ ^]^ Copyright 2018, Elsevier.

The piezoelectric effect is characterized by the coupling between mechanical strain and electrical polarization. As an intrinsic material's property, the piezoelectric effect permits simple architectures for mechanical energy harvesting compared to other electrostatic and electromagnetic mechanisms, which is particularly desirable for micro‐electro‐mechanical system (MEMS) devices and flexible systems. Generally, inorganic materials exhibit higher piezoelectric coefficients than organic materials. However, due to their high rigidity and brittleness, bulk inorganic piezoelectric materials are not amenable for use on soft biological surfaces. Thin films, membranes, ribbons, and nanowires are typical morphologies that researchers adapted to introduce high flexibility to the ceramic systems.^[^ ^45^ ^]^ High‐performance flexible PENGs have been demonstrated using representative lead‐based piezoelectric materials, e.g., Pb(Zr,Ti)O_3_ (PZT)^[^ ^46^, ^47^, ^48^ ^]^ and (1−*x*)Pb(Mg_1/3_Nb_2/3_)O_3−_ *_x_*PbTiO_3_ (PMN‐PT),^[^ ^49^ ^]^ with nanoscale structures. Although these materials possess excellent piezoelectric properties, they are not desired for being utilized in ecological/biological applications due to their long‐term toxicity. Lead‐free piezoelectric ceramics are a group of attractive alternatives to achieve high piezoelectric responses. A number of lead‐free piezoceramics, such as BaTiO_3_,^[^ ^50^ ^]^ (Bi,Na)TiO_3_,^[^ ^51^ ^]^ and BiFeO_3_‐based ceramics,^[^ ^52^ ^]^ have all been used in PENG development, however, with moderate performance due to their the low Curie temperatures and the high leakage levels. (K,Na)NbO_3_ (KNN)‐based materials recently have attracted significant attention as a high‐performance lead‐free piezoceramics because of their large piezoelectric coefficient, high Curie temperature, and good doping tunability.^[^ ^53^, ^54^, ^55^ ^]^ High‐performance KNN‐based PENG has been demonstrated in a large thin film morphology (**Figure** **2**a‐i). The polycrystalline KNN film was doped with Li ions and produced electricity that was compared to PZT‐based PENG. The ultrasmall thickness and good structural integrity yielded high flexibility, which allowed it to be conformally attached to a porcine heart for harvesting energy from heart beats.^[^ ^56^ ^]^

Implementation of piezoelectric polymers is another common approach to achieving highly flexible PENG devices. Additionally, polymer‐based PENGs often have lower weight compared to ceramic ones. A typical polyvinylidene fluoride (PVDF)‐based PENG is shown in Figure 2a‐ii. By introducing the mesoporous structure to PVDF films, Zheng et al. further lowered its mechanical modulus to the tissue level.^[^ ^57^ ^]^ Such a sponge‐like PVDF‐based PENG was able to generate appreciable piezoelectric voltage output following the muscle movements when implanted under the skin of living mice and rats.^[^ ^44^ ^]^ Although PVDF and its copolymer films (e.g., PVDF‐TrFE) have been broadly used as a polymeric piezoelectric component, its relatively low electromechanical coupling factors still resulted much lower electrical outputs compared to the ceramic‐based PENGs.^[^ ^43^, ^58^, ^59^ ^]^

Considering the special requirement of biocompatibility and biodegradability, biomaterials such as piezoelectric microorganisms and organics have also attracted much attention for implantable PENG development. Lee et al. designed a PENG assembled by bio‐based nontoxic virus (M13 bacteriophage), which was able to generate electricity of 6 nA and 400 mV under applied load of 34 N at a low frequency of 0.16 Hz.^[^ ^60^ ^]^ Similarly, Shin et al. prepared vertically aligned 4E‐M13 bacteriophage nanopillars arrays (Figure 2a‐iii) that could generate output voltage of ≈232 mV and current of ≈11.1 nA under an applied load of 30 N at 0.5 Hz.^[^ ^61^ ^]^ Application of phage molecules introduced a controllable biodegradability to PENG, which enabled the potential for applications as transient bioelectronics. Dipole alignment is essential, and also stands as a critical challenge for biomaterials to achieve high piezoelectric outputs. Recently, Wang et al. developed a confinement‐cell technology that led to large‐scale self‐assembly of vertical cellulose nanocrystals (CNCs), demonstrating the highest piezoelectric property compared to other CNC‐based films.^[^ ^62^ ^]^ Moving forward, more natural piezoelectric materials extracted from plants and animals, such as fish gelatin,^[^ ^63^ ^]^ biowaste fish skin,^[^ ^64^, ^65^ ^]^ diphenylalanine (FF) peptide, silk,^[^ ^66^, ^67^, ^68^, ^69^ ^]^ and bones are of substantial interests.

### 2.2 Triboelectric Nanogenerator

TENG is another mechanical energy harvesting principle relying on a combination of triboelectric effect and electrostatic induction.^[^ ^70^ ^]^ There are a number of insightful review articles describing different operating modes and principles of TENGs.^[^ ^71^, ^72^, ^73^, ^74^, ^75^ ^]^ Compared to piezoelectrics, the triboelectrification phenomenon exists in almost all materials, both natural and synthetic, ranging from metals to polymers, allowing a wide array of material choices for TENGs. Therefore, it is less challenging for TENGs to achieve desirable flexibility and biocompability in terms of material selections. A TENG generally consists of two sheets of films that exhibit distinctly different electron‐attracting abilities, with one electrophilic and the other electrophobic. Alternating potential difference will be induced upon cycled separation and re‐contact of the oppositely charged triboelectric materials. Therefore, the output performance of TENG is determined by the material properties including contact area, work function, electron affinity and permittivity.

An ultra‐stretchable micrograting sliding‐mode TENG represents a typical example for in vivo biomechanical energy harvesting, which converts the slow diaphragm motions in rats into high‐frequency alternating electricity based on the microscale electrode design (Figure 2b‐i).^[^ ^76^ ^]^ Packaged by a soft silicone elastomer with an internal cavity, the TENG device exhibited an ultralow Young's modulus of ≈45 kPa and a high compatibility to soft biological tissues. The TENG was implanted inside the abdominal cavity of Sprague Dawley (SD) adult rats and directly converted the mechanical energy from normal respiration into a continuous ≈2.2 V direct current (DC) output through a basic electrical circuit, which was able to continuously power a light‐emitting diode. This solely biomechanical energy‐driven DC micro‐power supply presents a promising solution for the development of self‐powered implantable medical devices.

Owing to the broad material selection, biodegradable TENGs have also received considerable development for the applications of transient implantable biomedical electronics.^[^ ^77^, ^78^, ^79^ ^]^ Biodegradable TENGs were typically fabricated from biodegradable polymers, such as PLGA, PHV/B, PVA, and PCL, and biodegradable metal electrodes, such as Mg, Mo, and Zn, in a multilayer structure for in vivo biomechanical energy harvesting (Figure 2b‐ii). The lifetime is normally controlled by the packaging layer. A 50 µm PLGA‐encapsulated TENG consisting of triboactive PLGA and PVA materials (2 cm × 3 cm) can degrade and be resorbed in the subdermal dorsal region of a SD rat after 9 weeks working cycle without any adverse long‐term effects.^[^ ^80^ ^]^ Other works introduce natural biodegradable material such as gelatin,^[^ ^81^ ^]^ silk,^[^ ^82^ ^]^ cellulose nanofibrils,^[^ ^83^ ^]^ and chitosan,^[^ ^76^ ^]^ as the functional material of TENGs to achieve different lifetime and electrical output. For instance, silk‐based TENG has a relative long degradation time of over 100 days in aqueous solution, whereas gelatin could quickly disintegrate in 20 days. Based on the electronic affinities in tribo‐series, pairing gelatin (negative) with polylactic acid, another biodegradable polymers (positive), could achieve an output as high as 400 V.^[^ ^46^ ^]^

Flexible materials design also allowed a switchable degradation that could be turned on by biophysical environments/parameters. As demonstrated by Li et al., gold nanorods were used as additives inside a biodegradable polymers matrix (Figure 2b‐iii).^[^ ^84^ ^]^ Under near infrared (NIR) illumination, the gold nanorods could generate heat and activate the degradation process. Without NIR, the device could sustain a relatively long time (28 days) of in vivo function. This is a highly preferred feature for transient biomedical electronics with variable operational time frames.

## 3 Electrodes for Biological Tissue Interfaces

In the field of bioelectronics, understanding of electronic communications between biology and electronics and developing suitable electrodes, known as bioelectronic interfacing, has been one of the critical research directions. The majority of existing and emerging bioelectronic interfaces involve various forms of electrode interacting with biological tissues.^[^ ^85^ ^]^ These interactions exist in all implantable bioelectronic systems that directly exchange electrical signal with human body, such as cochlear implants restoring hearing for people suffered from profound hearing loss, deep brain stimulation (DBS) alleviating Parkinsonian symptoms, spinal cord WIEs helping manage neuropathic pain, and vagal nerve neuromodulation alleviating depression. Compared to biological tissues, the electrode materials are typically several orders of magnitude rigid. Besides, tissues also display viscoelastic properties that are often anisotropic, withstand uninterrupted motion affected by different biological activities, and undergo changes in volume throughout life. Designing and fabricating electrodes that well interface with soft tissue and effectively deliver electrical signals still stands as one of the major challenges for the implementation of WIEs.

### 3.1 Microelectrodes and Arrays

**Figure 3:**
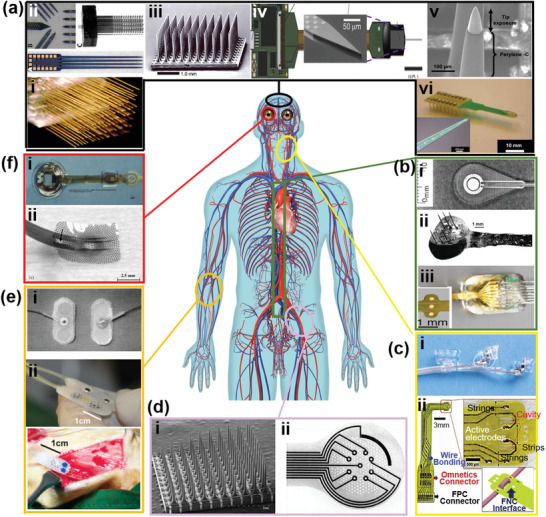
Rigid electrode designs for electroceuticals. a) Brain stimulation: i) Low‐density electrode array (NB Labs, Dennison, TX) consisting of 16–32 units of 50 µm Teflon‐coated stainless steel microwires. Reproduced with permission.^[^ ^87^ ^]^ Copyright 2003, National Academy of Sciences. ii) Michigan‐style Si‐based microelectrodes. Reproduced with permission.^[^ ^92^ ^]^ Copyright 2008, Society for Neuroscience. iii) Utah electrode array. Reproduced with permission.^[^ ^90^ ^]^ Copyright 2006, Nature. iv) Neuropixels probes based on Si array. Reproduced with permission.^[^ ^93^ ^]^ Copyright 2017, Springer Nature. v) Pt/Ti/W/Pt neuron probe coated with iridium oxide. Reproduced with permission.^[^ ^95^ ^]^ Copyright 2010, Institute of Physics. vi) MEA surface with (PEDOT) nanotubes. Reproduced with permission.^[^ ^96^ ^]^ Copyright 2008, Elsevier. b) Spinal cord stimulation: i) Electrode design with umbrella‐like anchors. Reproduced with permission.^[^ ^97^ ^]^ Copyright 2001, Elsevier. ii) Integrated stimulation microelectrodes array designed to float on the dorsal surface of the spinal cord. Reproduced with permission.^[^ ^98^ ^]^ Copyright 2001, Elsevier. iii) Microwire electrodes designed with computer simulation. Reproduced with permission.^[^ ^99^ ^]^ Copyright 2016, Springer Nature. c) Sciatic nerves stimulation: i) Slanted UEA to achieve better penetration. Reproduced with permission.^[^ ^105^ ^]^ Copyright 2009, Elsevier. ii) Hierarchical round‐shaped polyimide sieve electrodes. Reproduced with permission.^[^ ^106^ ^]^ Copyright 2017, Wiley‐VCH. d) Vagus nerve stimulation: i) A bifurcated helical lead‐based vagus nerve stimulation electrode. Reproduced with permission.^[^ ^101^ ^]^ Copyright 2004, IEEE. ii) A neural clip implant. Reproduced with permission.^[^ ^102^ ^]^ Copyright 2002, Wiley. e) Muscle stimulation: i) Two‐point electrode consisting of two conducting stainless steel pads with a helix polypropylene monofilament threaded through the center. Reproduced with permission.^[^ ^111^ ^]^ Copyright 1997, IEEE Computer Society. ii) A miniaturized multichannel electrode with silicone substrate. Reproduced with permission.^[^ ^112^ ^]^ Copyright 2015, Institute of Physics Publishing. f) Eye stimulation: i) Biomimetic implantable retinal electrode arrays. Reproduced with permission.^[^ ^114^ ^]^ Copyright 2009, Institute of Physics Science. ii) A polyimide packaged micromachined epiretinal vision prosthesis. Reproduced with permission.^[^ ^115^ ^]^ Copyright 2008, Elsevier.

Early electrodes in biomedical applications were single or multiple microwires fabricated with inert metals including stainless steel, tungsten, gold, and platinum, owing to their high conductivity and chemical stability in physiological environments.^[^ ^86^ ^]^ A paradigm is shown in **Figure** **3**a–i, a commercially available low‐density electrode array (NB Labs, Dennison, TX) consisting of 16–32 units of 50 µm Teflon‐coated stainless steel microwires was used for recording the activity of single cortical neurons from awake, active macaque monkeys.^[^ ^87^ ^]^ These electrodes have impedance between 100 kΩ and 1 MΩ at 1 kHz, which could stimulate brain at charge injection capacity from 0.1 to 1 mC cm^–2^.^[^ ^88^, ^89^ ^]^

With further advances in microfabrication technology, heavily doped Si‐based microelectrode array (MEA) was later used for bioelectronics.^[^ ^90^, ^91^ ^]^ The Michigan‐style Si‐based microelectrodes (Figure 3a‐ii) are able to place along single or multiple planar shanks to record feedback electric signals at well‐controlled tissue depth.^[^ ^92^ ^]^ Normann et al. developed an silicon‐based needle‐like alternative MEA, called Utah electrode array (UEA, Figure 3a‐iii), for brain stimulation.^[^ ^90^ ^]^ The UEA used glass reflow, dicing, and etching to create an array of well‐defined penetrating electrode tines. It is the only high‐density, penetrating recording electrode approved by the Food and Drug Administration (FDA) so far. The Si‐based electrode could also be fully integrated at a high density of 25 electrodes mm^–2^. Compared to conventional extracellular probes, the neuropixels probes based on Si array provided an order of magnitude increase in the number of simultaneously recorded neurons per shank while maintaining low noise (Figure 3a‐iv).^[^ ^93^, ^94^ ^]^ Owing to the integrated circuitry for amplification, multiplexing, and digitization, this probe had a small physical footprint and minimal cabling, requiring only a simple interface board to acquire data, which was important for studying unrestrained behavior. In order to achieve higher charge injection capacity and lower impedance, Pt/Ti/W/Pt coated with iridium oxide (Figure 3a‐v) have also been used instead of the gold or platinum contacts on the terminal recording sites.^[^ ^95^ ^]^ To improve the electrode–tissue interface both electrically and mechanically, poly(3,4‐ethylenedioxythiophene) (PEDOT) nanotubes have been electrochemically polymerized on the surface of MEA (Figure 3a‐vi), and reduced the impedance by almost two orders of magnitude at a frequency of 1 kHz.^[^ ^96^ ^]^

Besides brain stimulations, MEA continued to thrive as regenerative electrode and therapeutic option for spinal cord and nerve injured patients. First generation of spinal cord stimulation was achieved by external power transmitter and implanted inert electrodes (platinum or iridium). Small, umbrella‐like anchors are attached to the tip to hold the electrode in the tissue (Figure 3b‐i). With this arrangement, the diameter of the electrode tip did not differ much from the lead‐wire diameter, and this electrode could be introduced into a deep muscle with a trocar‐like insertion tool.^[^ ^97^ ^]^ In a further development, integrated stimulation microelectrodes array (Figure 3b‐ii) was designed to float on the dorsal surface of the spinal cord, an approach that should be well adapted to the large subdural space above the human sacral cord. The electrodes were able to penetrate into the cord at a moderately high velocity (≈1 m s^−1^).^[^ ^98^ ^]^ Another microfabricated microwire electrodes for spinal cord treatment was designed with computer simulations, which enable tailoring of the topologies and dimensions of the implants according to the anatomy of the spinal cord and vertebra structures (Figure 3b‐iii).^[^ ^99^ ^]^ In order to accommodate to the cylinder geometry of spinal cord and nerve bundles, a panoply design, such as cuff, intra‐fascicular, shaft and regenerative electrodes has been introduced and tested.^[^ ^100^ ^]^ As shown in Figure 3c‐i, the UEA was changed into a slanted one to achieve better penetration for sciatic nerves stimulations.^[^ ^101^ ^]^ Others demonstrated that a round shape polyimide sieve electrodes with 281 round via‐holes (40 µm in diameter) and nine integrated stimulating electrodes arranged around the via‐holes (Figure 3c‐ii). This hierarchical structure allowed implantation between the severed ends of the sciatic nerve and successfully helped nerve regeneration.^[^ ^102^, ^103^, ^104^ ^]^ The cuff and intra‐fascicular interfaces are invasive, leading to potential nerve damage. They are also difficult to implant on small visceral nerves. For the use of extra‐neural cuff electrodes, Figure 3d‐i shows a typical vagus nerve stimulation electrode, a bifurcated helical lead, to which a pulse generator delivers electrical signals to realize neuromodulation of the cervical vagus nerve trunk.^[^ ^105^ ^]^ Lee et al. proposed a neural clip implant (Figure 3d‐ii) that enabled conformal implantation on a variety of small peripheral nerves.^[^ ^106^ ^]^ They demonstrated that this clip‐like electrode could well accommodate to the vagus, bladder, and the sciatic nerve branches for modulation and stimulation.

For muscle and eye stimulations, the structures of electrodes were also designed preferentially according to the curvilinear shape. Commercial single‐point stimulation electrode has been the most frequently used electrode for muscle reviving therapy. The structure was relatively simple, consisting of only conducting central disk (stainless steel) and leads wire.^[^ ^107^, ^108^, ^109^, ^110^, ^111^ ^]^ This single‐point stimulation electrode further evolved to a two‐point electrode, consisting of two conducting stainless steel pad with a helix polypropylene monofilament threaded through the center (Figure 3e‐i).^[^ ^111^ ^]^ Silicone was also been used for planar electrodes with a miniaturized multichannel electrodes to offer enhanced flexibility (Figure 3e‐ii).^[^ ^112^ ^]^ This electrode was implanted in the muscles of interest (e.g., biceps, triceps, lateral deltoid) for muscle activity recording and stimulation.^[^ ^105^, ^113^ ^]^ Implantable retinal electrode arrays were designed using 75 µm‐diameter electrodes arranged in a complex biomimetic pattern that closely mimics the density of ganglion cells in the human retina (Figure 3f‐i). They were heat molded to the approximate curvature of the canine retina.^[^ ^114^ ^]^ To achieve smooth interfaces preventing nerve trauma by sharp edges and corners, Stieglitz et al. utilized polyimide as the encapsulation material, and fabricated a micromachined epiretinal vision prosthesis (Figure 3f‐ii).^[^ ^115^ ^]^ Nevertheless, each of them was rigid to eyeball since the inner components were still bulky metals with unrefined structure.

Though these MEAs could provide high spatial resolution, large injection currents with high signal‐to‐noise ratio for stimulation applications, the geometry innovations did not supersede conventional microwire designs in evading host immune responses.^[^ ^116^, ^117^, ^118^ ^]^ Activation of immune responsive cells could generate an insulation layer or sheath around the electrodes. The sheath could become increasingly denser in 6 weeks following implantation, and as a result, the impedance of the electrode‐issues interface would increase, which could significantly jeopardize stimulation effectiveness.^[^ ^118^ ^]^ Aware of this, two strategies were proposed to reduce the immune response: 1) to coat hyaluronic acid or polyethylene glycol (PEG) containing hydrogels to control hydrophobicity of the electrodes^[^ ^119^, ^120^ ^]^ and 2) to bind bioactive molecules on the surface of electrodes.^[^ ^120^, ^121^ ^]^ However, both methods are time‐limiting and could not solve the problem of stiffness mismatch between the soft tissues and the electrodes.

### 3.2 Advanced Stretchable and Conformable Electrodes

As the stimulation electrodes evolved into 2D arrays, they imposed much high requirements toward the flexibility and stretchability. Tissue surfaces are soft with irregular and complex topography. The brain, as the most complex biological organ, displays complex external topographies and patterns of convolution that result from gyrification of the cortical surface.^[^ ^122^ ^]^ Typical reported elastic moduli range from a few 100 Pa to about 10 kPa, depending on strain, shear rate, preconditioning, and several other factors.^[^ ^123^ ^]^ Therefore, in order for a 2D electrode array to achieve conform contact, stretchability is the first main factor to consider systematically. Implant (macroscopic) compliance may be achieved via the use of ultrathin materials. The flexural stiffness, *D*, of a material is defined in Equation (1): the thinner the material, the more flexible it is^[^ ^124^ ^]^

(1) $$D=\frac{Eh^{3}}{12\left(1-v^{2}\right)}\;$$

where *E* is the elastic modulus, *h* is the thickness of the material, and *ν* is Poisson's ratio.

**Figure 4:**
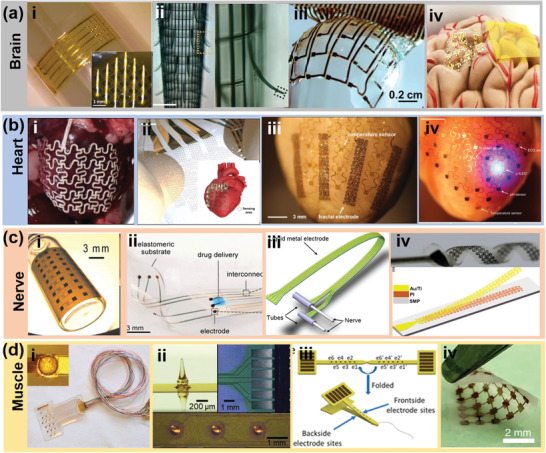
Flexible and conformal electrodes for electroceuticals. a) Electrodes on brain: i) A thin‐film flexible mesh substrate with microneedle electrode. Reproduced with permission.^[^ ^125^ ^]^ Copyright 2016, Nature. ii) A penetrating electrode utilizing a cylindrical macroporous structure. Reproduced with permission.^[^ ^126^ ^]^ Copyright 2015, Springer Nature. iii) Non‐penetrating ultrathin and flexible electrodes combined with micrometer‐thick silicon transistor membranes. Reproduced with permission.^[^ ^78^ ^]^ Copyright 2010, Springer Nature. iv) Green electrodes with zero footprint. Reproduced with permission.^[^ ^127^ ^]^ Copyright 2017, United States National Academy of Sciences. b) Electrodes on heart: i) An electric mesh wrapping around the heart. Reproduced with permission.^[^ ^128^ ^]^ Copyright 2016, American Association for the Advancement of Science. ii) An ultrathin, stretchable honeycomb electrode with conformal contact to heart surface. Reproduced with permission.^[^ ^129^ ^]^ Copyright 2018, American Association for the Advancement of Science. iii) A combined fractal design constructed from the second‐order iteration of the Greek cross fractal motif with serpentine traces. Reproduced with permission.^[^ ^130^ ^]^ Copyright 2015, Wiley‐VCH. iv) A conformal elastic membrane with 3D printing technologies to match the epicardium of heart. Reproduced with permission.^[^ ^131^ ^]^ Copyright 2014, Nature. c) Electrodes wrapping nerve: i) A cylinder‐shaped electrode by inkjet printing. Reproduced with permission.^[^ ^132^ ^]^ Copyright 2019, American Association for the Advancement of Science. ii) A hook‐shaped electrode. Reproduced with permission.^[^ ^133^ ^]^ Copyright 2015, American Association for the Advancement of Science. iii) A flexible and stretchable neural cuff electrode. Reproduced with permission.^[^ ^135^ ^]^ Copyright 2017 Institute of Physics. iv) A type of twining electrode. Reproduced with permission.^[^ ^136^ ^]^ Copyright 2019, American Association for the Advancement of Science. d) Electrodes on muscle: i) A stretchable multielectrode array by embedding patterned Au electrodes in a PDMS substrate. Reproduced with permission.^[^ ^138^ ^]^ Copyright 2011, IEEE. ii) A penetrating five‐point epimysial electrode. Reproduced with permission.^[^ ^139^ ^]^ Copyright 2019, Wiley‐VCH. iii) A 2D ribbon‐like multichannel electrode design. Reproduced with permission.^[^ ^141^ ^]^ Copyright 2019, American Chemical Society. iv) A holey structure electrode design with both Si mesh membrane and PDMS substrate. Reproduced with permission.^[^ ^142^ ^]^ Copyright 2018, Springer Nature.

To accommodate the wrinkled brain surface, a microneedle electrode array was fabricated on a thin‐film flexible mesh substrate (**Figure** **4**a–i).^[^ ^125^ ^]^ Due to the sufficient stiffness of the electrode and the good flexibility of the mesh substrate, the electrode can penetrate into the tissue with its bottom layer fully conformal to the curved brain surface. Another penetrating electrode utilized a cylindrical macroporous structure (Figure 4a‐ii).^[^ ^126^ ^]^ It could penetrate brain tissue after rapid freezing in liquid nitrogen with minimal invasive injury. It can recover the high flexibility in the brain tissue after insertion, which was an impressive mechanical advantage compared to the microneedle electrodes. A non‐penetrating electrode (Figure 4a‐iii) used ultrathin and flexible electrodes combined with micrometer‐thick silicon transistor membranes. It allowed intimate contact with inaccessible cortical areas such as the interhemispheric fissure.^[^ ^78^ ^]^ In addition to conformal adaptation, “green” electrodes with zero footprint (Figure 4a‐iv) were developed using a biodegradable ultrathin (800 nm) substrate based on cellulose. It could be completely disintegrated after 30 days upon exposure to mildly acidic conditions.^[^ ^127^ ^]^

Heart is another organ that stimulation electrodes are often applied on. To accommodate the dynamics of heart, the tensile stiffness, i.e., the ability of the electrodes to reversibly expand and relax with the underlying tissue, becomes a crucial design parameter. Traditional pacemaker electrodes just apply electrical stimulations at specific contact points and do not cover heart surfaces. In order to deliver electrical impulses to the whole ventricular myocardium, Park et al. devised an electric mesh (Figure 4b‐i) that could wrap around the heart.^[^ ^128^ ^]^ The mesh was made from silver nanowires embedded in a rubber polymer that could conform to the unique 3D anatomy of hearts. A serpentine mesh design was used with elasticity (elastic modulus was 44.71 ± 7.54 kPa) nearly identical to that of epicardium within a physiological strain range of 20% in cardiac movement. An ultrathin, stretchable honeycomb electrode (Figure 4b‐ii) was designed to achieve conformal contact between the electrode and the heart surface.^[^ ^129^ ^]^ The electrode had a total thickness of only 2.6 µm and a high average transconductance (gm) of 1 mS, which could largely decrease the motion artifact noise. Xu et al. demonstrated a combined fractal design, constructed from the second‐order iteration of the Greek cross fractal motif with serpentine traces that filled a rectangular area (Figure 4b‐iii).^[^ ^130^ ^]^ The Greek cross involved a high degree of geometrical connectivity, which was effective to reduce the electrical resistance while providing a high degree of tolerance to defects. This design enabled non‐uniform current distribution on the electrode, where additional charges distributed at the electrode edges. Compared to a conventional electrode, this design largely increased the ratio of electrode–insulation edge length to the geometric surface area of the electrode. This feature suggested that a fractal electrode design should transfer current more efficiently from the electrode to the tissue. Such improvements were particularly beneficial to the development of low power systems. By combining a conformal elastic membrane with 3D printing technologies, the electrode was precisely shaped to match the epicardium of heart.^[^ ^131^ ^]^ This electrode configuration (Figure 4b‐iv) could establish a conformal interface with the epicardium and perform a variety of high‐density physiological multiparametric stimulation. These approaches present a promising opportunity to design and implement high‐definition implantable devices for treating lethal heart diseases.

For peripheral nervous system (PNS) interfaces, the shape of the devices is particularly important to alleviate demyelination and nerve loss for long‐term neural stimulation and recording. A cylinder shaped electrode with patterned interdigitated MEAs was fabricated by inkjet printing on a polyimide substrate with a bending radius of 14 mm, as shown in Figure 4c‐i.^[^ ^132^ ^]^ This approach allowed fine silver microwires (≈10 µm) to be bonded with minimal contact pressure on both flat and curved surfaces, which was an improvement as compared to traditional nervous electrodes. A hook shape electrode for nerve was shown in Figure 4c‐ii. It integrated a transparent silicone substrate (120 µm), stretchable gold interconnects (35 nm), and soft electrodes coated with a platinum‐silicone composite (300 µm).^[^ ^133^ ^]^ The hook structure was less constraint and more flexible compared to the cylinder electrodes. However, peripheral nerves are anisotropic structures that spread throughout the body with varied diameters. For example, the small visceral nerves are about 100 times smaller than the sciatic nerve, which reaches up to 2 cm in diameter in humans. As the tensile loading increases, the nerve displays quasi‐elastic stress–strain behavior until rupture of the perineurial sheath, which occurs at a fracture strain of ≈25% and an ultimate stress of ≈10 MPa.^[^ ^134^ ^]^ Therefore, researchers began to investigate cuff electrodes, which could be deposited on an ultrathin substrate and then rolled onto a cylindrical‐shaped nerve. This configuration could minimize the potential damage to the peripheral nerve while attaining high quality signal. In Figure 4c‐iii, a flexible neural cuff electrode embedded with liquid metal electrodes and stretchable interconnects in a PDMS membrane (500 µm in thickness) was shown. The whole device had a low elastic modulus (1.055 MPa) that was similar to neural tissues (0.1–1.5 MPa).^[^ ^135^ ^]^ Electrical measurements revealed that the liquid metal interconnects could sustain over 7000 mechanical stretch cycles with a stable resistance of 4 Ω. Zhang et al. proposed another type of twining electrode (Figure 4c‐iv) by integrating stretchable mesh serpentine wires onto a flexible shape memory substrate. Involving shape memory components offered permanent shape reconfigurability, distinct elastic modulus controllability (from ≈100 MPa to ≈300 kPa), and shape recoverability at body temperature. Similar to the climbing process of twining plants, a flat 2D stiff twining electrode could naturally self‐climb onto nerves in the presence of a 37 °C saline solution, and thus forms 3D flexible neural interfaces with minimal constraint on the deforming nerves.^[^ ^136^ ^]^

As skeletal muscle has a relative flat surface but also widely distributed motoneurons, researches on muscular stimulation electrode are mostly focused on flexibility, stable power delivery and multi‐point stimulation. As shown in Figure 4d‐i, a stretchable multielectrode array for spatiotemporal epimysial stimulation was fabricated by embedding patterned Au electrodes in a PDMS substrate.^[^ ^137^, ^138^ ^]^ To increase the adhesive ability and effective delivery, Wang et al. integrated a penetrating five‐point epimysial electrode with Au coated SU‐8 spikes (Figure 4d‐ii).^[^ ^139^ ^]^ During muscle contractions, the conventional flat epimysial electrodes were vulnerable to detachment from muscle surface. The spiked epimysial electrode could remain in the muscle tissue and be tightly surrounded by muscle fibers to accommodate muscle structure change during contractions. In addition, the threshold charge for directly eliciting muscle fiber action potentials was much greater than the threshold for producing action potentials in motoneurons. As a result, electrical muscle stimulation typically requires mA‐level stimulation current,^[^ ^140^ ^]^ which necessitates muscle electrode designs to access the sparsely distributed motoneurons to optimize stimulation efficiency. In aware of this, Wang et al. further proposed a 2D ribbon‐like multichannel electrode design by depositing highly conductive Au thin films onto patterned flexible polyimide thin films (Figure 4d‐iii).^[^ ^141^ ^]^ Alternatively, Jiang et al. utilized a distributed mesh of Si membrane (≈2.3 µm in thickness) and a porous polydimethylsiloxane (PDMS) substrate (≈120 µm in thickness) in electrode design (Figure 4d‐iv). The holey structure in both Si and PDMS could mitigate the stress accumulated across a large area and thus enhanced the electrodes’ mechanical compliance, which was much alike the principle of the mesh electrodes for heart applications.^[^ ^142^ ^]^

## 4 WIE Applications

ES has an intriguing history of neuromodulation such as cochlear implants, DBS and spinal cord pain control. To date, several clinical trials have led to successful FDA Pre‐Market Approvals, including the Second Sight Argus II System to restore patients’ vision, the NeuroPace Responsive Neural Simulation System to treat intractable epilepsy, the Inspire Airway Stimulation System to treat obstructive sleep apnea, the Enteromedics VBLOC vagal blocking system for treating obesity, and the CVRx Rheos baroreceptor activation therapy to treat intractable hypertension. These clinical promises have motivated a new era of research on underlying bioelectronic biology, therapeutic mechanisms, and safe and efficacious stimulation protocols. From engineering and materials perspectives, innovations on flexible, wearable, and implantable ES devices remain as the core of research to provide convenient, highly accessible, and minimal invasive ES therapies. In this section, we review representative applications of state‐of‐the‐art ES devices in different therapeutic areas.

### 4.1 Wearable and Implantable Pacemakers

The basic function of heart, i.e., heartbeat, is triggered by intrinsic electrical pulses, which control the alternative contraction and relaxation of atria and ventricles. Specifically, it starts with the natural pacemaker cell (sinoatrial node, SA node) located in the right atrium producing electrical pulses, which spread through the walls of both right and left atria and cause them to contract. This electrical signal further travels through atrioventricular node (AV node) to His–Purkinje network of both ventricles and trigger their contraction accordingly. When this electrical system is not functioning properly due to the irregular electrical generation or transportation, the heart rate and rhythm of the heartbeat would become abnormal (arrhythmia).

The most common wearable and implantable device for heart stimulation is an artificial pacemaker. A modern pacemaker consists of a small pulse generator implanted under the skin of the chest and leads that enter the venous system through upper extremity veins. The leads could be positioned in different chambers of the heart, depending on medical conditions. Cardiac pacing is determined by the programming of pacemaker, i.e., demand pacing and rate‐responsive pacing. While a demand pacemaker monitors heart rhythm and sends electrical pulses under abnormal heart rhythm, a rate‐responsive pacemaker will adapt the heart rate to the activity level by monitoring factors such as sinus node rate, breathing, and blood temperature.

Most commercial pacemakers are powered by primary battery with a typical energy consumption of 10–15 µW. From the beginning with nickel–cadmium battery in 1958 to the current lithium/carbon monofluoride (Li/CF*_x_*) battery, the capacity and stability of pacemaker battery has been significantly improved.^[^ ^11^, ^143^, ^144^ ^]^ However, to ensure a reasonably long operational lifetime (such as 7 years of full pacing), the battery component still has to make up to 80% or more of the volume and weight of the entire device, severely impeding its miniaturization and flexibility. Moreover, a retrieval procedure is eventually needed to replace the battery when the capacity drops to a critical level, which increases risk of inflections and complications.^[^ ^145^, ^146^ ^]^

To solve the issues of battery capacity, small rechargeable batteries with in vivo charging ability have been proposed. The wireless in vivo charging could be achieved through three approaches, namely, electromagnetic energy transfer, ultrasound energy transfer, and optical energy transfer. Despite some encouraging progresses, battery charging relies on patients’ ability/memory, which could introduce unnecessary risk to the life‐threatening situations. Besides, other limitations such as small transferring distance, low charging efficiency, strong tissue attenuation, and potential tissue damages also restrict the practical application of rechargeable batteries.^[^ ^147^, ^148^ ^]^ Nuclear batteries have also been proposed as another alternative power source for pacemakers. However, the toxic and radioactive fuel remains a major concern for the application.^[^ ^149^ ^]^

Most recently, evolution of NG technology has attracted intensive attention as a promising alternative power source to eliminate rigid bulky batteries and might significantly improve the device softness and flexibility. Given that the mechanical power from many body motions is at the milliwatt to watt level, theoretically, it is feasible to enable the self‐powering capability of cardiac pacing system which only requires microwatt‐level power. Hitherto, promising advances have been made in self‐powered cardiac system, particularly with the aids of soft and flexible TENGs and PENGs. Two strategies have been adopted to design the TENG/PENG‐based cardiac system, i.e., the indirect stimulation and direct stimulation.

In the models of indirect stimulation, the TENG/PENG works only as a power source to support the operation of a pacemaker. A TENG‐drive pacemaker was first demonstrated by Zheng et al.^[^ ^150^ ^]^ This self‐powered pacemaker system was composed of a TENG, a rectifier, a capacitor, and a pacemaker prototype. The TENG was implanted under left chest skin of a rat. It was able to convert energy from breathing into ≈±3 V AC electricity, which would be transformed by the rectifier and stored in a capacitor to drive the pacemaker prototype. A total of 13 750 breathing cycles could charge the capacitor from 2 to 3 V within 275 min. This amount of energy was sufficient for the pacemaker to generate heart stimulation pulses at various frequencies from 2 to 5 Hz.

**Figure 5:**
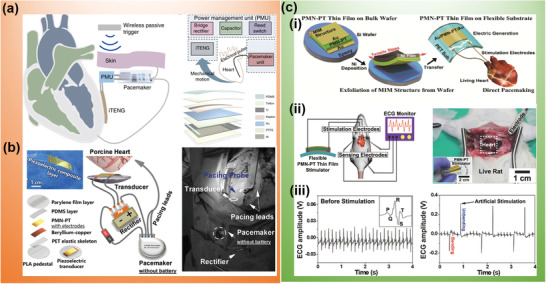
Functional WIEs for heart stimulation. a) Indirect cardiac stimulation enabled by a symbiotic system consisting of a TENG as power source, a PMU, and a pacemaker. Reproduced with permission.^[^ ^151^ ^]^ Copyright 2019, Springer Nature. b) Indirect cardiac stimulation enabled by a high‐power PMN‐PT‐based PENG activating up pacemaker by harvesting porcine heart motion. Reproduced with permission.^[^ ^155^ ^]^ Copyright 2020, Wiley‐VCH. c) Direct heart stimulation by a TENG performed under in vitro condition: i) Schematics of the PMN‐PT‐based PENG for direct pacemaking. ii) Schematics and real scenario of using PENG stimulating rat heart. iii) Comparison of rat ECG amplitude before stimulation and under artificial stimulation. Reproduced with permission.^[^ ^49^ ^]^ Copyright 2014, Wiley‐VCH.

Instead of using mechanical energy from chest motion, a symbiotic pacemaker driven by a porcine heart was developed by Ouyang et al, as shown in **Figure** **5**a.^[^ ^151^ ^]^ The system consisted of a TENG, a power management unit (PMU) and a pacemaker. Energy harvested by the TENG would be stored in the PMU first, and once it reached the desired value, the stored energy would activate the pacemaker. In this work, the TENG had a core–shell structure with nanostructured polytetrafluoroethylene and aluminum thin film as the triboelectric pair. It was implanted on the left ventricle between the heart and pericardium. At a heart rate of ≈82 bpm, the implanted TENG generated a voltage of 39 V and a current of 0.8 µA, equivalent to 0.495 µJ output energy, which was higher than the endocardial pacing threshold energy of 0.377 µJ. Nevertheless, 1 hour consistent operation of the implanted TENG could only charge the pacemaker for 2 min stimulation for heart rhythm correction. The required significantly longer charging time compared to the stimulation time was the main concern for practical applications. To avoid the invasive thoracotomy and relevant complications, the miniaturized and flexible TENG‐based WIEs also exhibit promises for intracardiac stimulation through a less invasive transcatheter implantation procedure.^[^ ^152^ ^]^

Compared to TENG devices, advanced piezoelectric materials with excellent electromechanical coupling property allowed for significantly boosted power.^[^ ^153^, ^154^, ^155^ ^]^ A (72%)Pb(Mg_1/3_Nb_2/3_)O_3_‐(28%)‐PbTiO_3_ (PMN‐PT) with exceptional *e*_33_ up to 18.03 C m^–2^ was exploited for building a bow‐shaped PENG. The PMN‐PT layer (50 µm) was sandwiched between a thin metal sheet and polymer layers to achieve desired flexibility. This device yielded an in vitro output power of 6.9–33 µW (Figure 5b). When implanted into the pericardial sac of an adult Yorkshire swine, the PENG was periodically compressed and relaxed by cardiac apex, yielding a *V*_oc_ and *I*_sc_ of 8.1 V and 30 µA, respectively. Through a rectifier, the AC output of the PENG was converted to a DC signal, which could directly power the pacemaker circuit bypassing any energy storage modules. The pacing impulse voltage of pacemaker driven by PENG stabilized at 3.0 V with a time interval of 0.85 s and an impulse signal width of 2 ms, demonstrating its full function of heart pacing and rhythm regulation. Another soft PENG was built from helical porous PVDF‐TrFE thin films, allowing it to be seamlessly integrated with pacemaker leads. Driven by bending and twisting motion of the heart, this PENG demonstrated a potential of powering the pacemaker for cardiovascular stimulation.^[^ ^156^, ^157^ ^]^

Compared to above indirect stimulations, direct stimulation is a more straightforward approach where the TENG/PENG could function as both power source and the pacing system. This strategy was demonstrated by Hwang et al. in 2014 when they used a PENG to stimulate the heart of rat directly through two electrode leads (Figure 5c).^[^ ^49^ ^]^ A PMN‐PT thin film was attached to a PET film to form a flexible membrane‐like PENG. Under a bending test, its output voltage and current reached up to 8.2 V and 145 µA, respectively. The corresponding energy under each bending cycle reached 2.7 µJ, which was higher than the threshold of triggering heart contraction. Therefore, the instantaneous electric pulses were used to directly stimulate a rat heart without any external power sources or electronic circuits. Before stimulation, the rat had a typical QRS complex with a fast heart rate of 6 Hz. After receiving the simulation from PENG, the heart rate was regulated down to 1 Hz with a regular heartbeat electrocardiogram (ECG) pattern, indicating a normal pacing function. However, this demonstration was only operated in vitro where the PENG was activated externally by direct pressing. What to use as an appropriate mechanical energy source to achieve effective in vivo stimulation remains the biggest question.

### 4.2 Electroceuticals for Neuromodulation

Neuromodulation denotes controlled ES of the central or PNS. Three forms of neuromodulation, including sciatic nerve stimulation, vagus nerve stimulation, and DBS, will be discussed in this section as representative demonstrations for wearable/implantable electroceutical applications.

Traditional neuromodulators powered by batteries typically use “square‐wave” pulses as the stimulation signal.^[^ ^158^ ^]^ Other studies also proposed that “exponential wave” might realize a more effective neuron stimulation with a lower power consumption.^[^ ^159^, ^160^ ^]^ The electrical outputs from NGs (both TENGs and PENGs) also exhibit a similar exponential waveform (or degenerate wave), allowing NGs to be directly used for neurostimulation. In 2012, TENGs were demonstrated to be able to successfully stimulate frog's sciatic nerve in vitro.^[^ ^161^, ^162^, ^163^ ^]^ The sciatic nerve were directly actuated by the high‐voltage electric pulses directly from the TENG device (58–209 V). Lee et al. further investigated the stimulation efficiency of TENG on rat's sciatic nerve and pelvic nerve.^[^ ^164^, ^165^ ^]^ They confirmed that exponential waves could achieve higher nerve stimulation efficiency than square pulse waves. Even though the current amplitude (200 µA) and the amount of charge (10–26 nC) were all lower than the threshold level required for neuron stimulation by square wave current (500 µA), highly efficient activations were still achieved. However, this could not be well explained by the well‐known charge‐based nerve stimulation theory. Since lower charges could reduce the risk of stimulation‐induced tissue and electrode damages, NG‐driven wearable and implantable neuromodulators may offer unique advantages for ES of nerves.

Vagus nerve stimulations have a relatively shorter history than other nerve stimulation methodologies. However, since the first hypothesis of potential application to refractory epilepsy in the 1980s, the clinical development has been quite fast. Clinical studies have demonstrated that vagus nerve stimulation could induce multiple physiologic functions related to refractory epilepsy, depression, anxiety, obesity, migraine, and Alzheimer's disease.^[^ ^166^, ^167^ ^]^ Commercial vagus nerve WIEs, consisting of electrodes, a pulse generator/battery, and a lead, can provide well controlled ESs, which are intermittent (e.g., 30 s stimulation every 5 min) and relatively safe but still accompanied with several side effects such as slight pain, burning, tingling, or itching sensation under the electrodes.^[^ ^168^ ^]^

**Figure 6:**
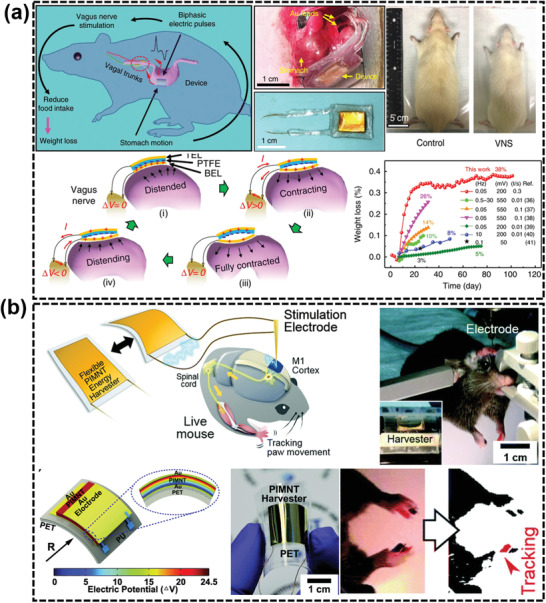
WIEs for nerve stimulation. a) A WIE driven by stomach peristalsis providing pulsed electricity to rat vagus nerve system to regulate its weight. The treated rat group within 100 days showed 38% less weight than the control group. Reproduced with permission.^[^ ^169^ ^]^ Copyright 2018, Springer Nature. b) A single‐crystalline PIMNT‐based WIE enables real‐time DBS of the mice motor cortex by providing high current reaching 0.57 mA through slight bending. The M1 cortex in the brain was successfully activated by electrodes directly connected to the WIE device, and thereby could efficiently induce forearm movements in mice. Reproduced with permission.^[^ ^183^ ^]^ Copyright 2015, Royal Society of Chemistry.

As TENG has demonstrated its advantage in neuromodulation of sciatic nerve, Yao et al. developed a presentative battery‐free vagus nerve WIE for weight control based on an implantable TENG device on stomach surface. It could spontaneously respond to stomach movements and generate electrical pulses toward vagus nerves to reduce food intake and thus control obesity.^[^ ^169^ ^]^ As shown in **Figure** **6**a, the flexible and biocompatible TENG was sew to the stomach outer surface, generating biphasic electrical pulses in responsive to the peristalsis of stomach. The electrical pulses were then transmitted to the anterior vagus nerves and posterior vagus nerves via two leads. By this strategy, the average body weight of rats was controlled at 350 g, 38% less than the untreated groups. This work correlated nerve stimulation with targeted organ functionality through a smart, self‐responsive system forming a closed loop of both energy flow and function feedback. This NG‐enabled closed‐loop ES may offer a new and more effective therapeutic technology using artificial electrical signals generated from coordinated body activities.

DBS is an established treatment for severe movement disorders such as Parkinson's disease,^[^ ^170^ ^]^ tremor,^[^ ^171^ ^]^ Tourette syndrome,^[^ ^172^ ^]^ pain,^[^ ^173^ ^]^ dystonia,^[^ ^174^ ^]^ depression,^[^ ^175^ ^]^ and obsessive compulsive disorder.^[^ ^176^ ^]^ DBS modulates pathological activity within central neural networks with applied electric fields, with a fundamental effect of stimulating axons around the electrode.^[^ ^177^ ^]^ After more than three decades of clinical empiricism, a better understanding of DBS mechanisms from physiological research is now being translated into novel technical solutions. Researchers found that the activation threshold of neural elements with different membrane excitability properties varies with stimulus strength and pulse duration, and is reflected by the nonlinear strength duration or chronaxie relationship. Experimental data suggested potential benefits of shorter pulse durations in increasing the therapeutic window, reducing capsular side effects of stimulation, and improving patients’ tolerance without sacrificing stimulation efficacy.^[^ ^178^, ^179^ ^]^ Computational models also suggested that non‐rectangular shape may improve the efficiency of DBS.^[^ ^180^, ^181^, ^182^ ^]^ Exploring safer technologies that can also be more therapeutic and energy efficient is the key of DBS device development.

NG has shown great promises to release DBS systems from battery support by introducing a self‐powered capability. Hwang et al. developed a PENG using a single crystalline Pb(In_1/2_Nb_1/2_)O_3_–Pb(Mg_1/3_Nb_2/3_)O_3_–PbTiO_3_ (PIN–PMN–PT:PIMNT) with a very high piezoelectric coefficient (*d*_33_ ≈ 2700 pC N^–1^),^[^ ^183^ ^]^ as shown in Figure 6b. Through mechanical bending, the PENG generated a current of ≈45 µA at a load resistance of 200 kΩ, equivalent to the load impedance of electrodes in practical DBS. To determine the effect of stimulation, the M1 cortex in the brain was stimulated by electrodes directly connected to the PENG device. The periodic muscle contractions controlled by the M1 cortex evidenced the effectiveness of electrostimulation. This work successfully demonstrated the feasibility of using PENG to achieve self‐powered electroceutical for DBS.

### 4.3 Electroceuticals for Skin Regeneration

The epithelial layer uses endogenous electrical field, along with chemotaxis to guide epithelial cell migration for wound healing.^[^ ^184^, ^185^ ^]^ External ES has been shown having positive effects throughout the entire wound healing process, such as decreasing edema around the electrode, lysing or liquifying necrotic tissue, stimulating growth of granulation tissue, increasing blood flow, causing fibroblasts to proliferate and making collagen, inducing epidermal cell migration, attracting neutrophils, stimulating neurite growth directionally, promoting epithelial growth and organization, decreasing mast cells in healing wounds, attracting macrophages, and stimulating receptor sites to accept certain growth factors.^[^ ^186^ ^]^ Furthermore, low‐intensity ES therapy is safe with minimal side effects. It offers a unique, simple, and inexpensive intervention option to heal complicated and recalcitrant wounds.^[^ ^187^ ^]^

Several different modalities of ES have been applied for wound healing, including DC, alternating current (AC), high‐voltage pulsed current, low‐intensity direct current, and electrical biofeedback signals. Despite variations in the format, duration, and dosing of electrical signals, all of them showed appreciable improvements in wound healing rate and improved local perfusion compared to standard care situations or sham therapy. However, it is still not clear which one would offer the most optimal treatment for cutaneous wound healing.^[^ ^188^ ^]^ A few investigators suggested that compliance may be a factor that affects wound healing in ES treatments.^[^ ^189^ ^]^ Hypothetically, ES that can be consistently applied but with less electrical energy may be more effective with less side effects.

**Figure 7:**
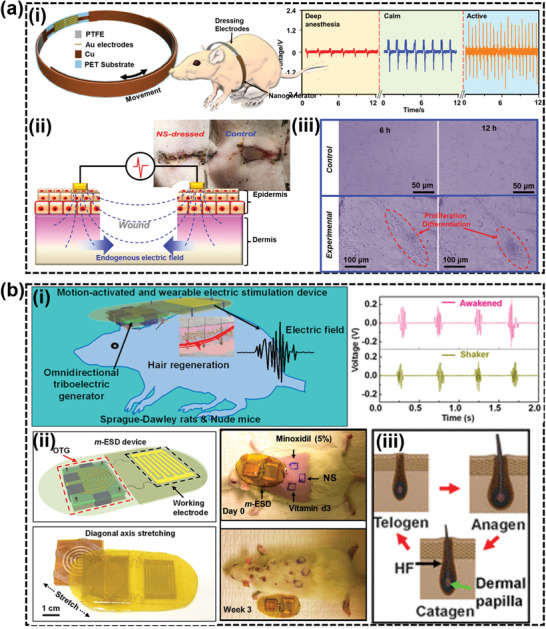
WIEs for epidermal stimulation. a) Accelerated wound healing through WIE: i) Schematics of WIE driven by rat breathing and the device outputs at different breathing frequency. ii) Wound healing accelerated with WIE stimulation and the behind mechanism. iii) NG‐based WIEs producing electric fields facilitating fibroblast cells growth, migration, and alignment. Reproduced with permission.^[^ ^190^ ^]^ Copyright 2018, American Chemical Society. b) Enhanced hair growth by WIE: i) Schematic of WIE for rat hair regeneration and device outputs through rat motions. ii) The WIE device and its 3 week impact on rat hair growth. iii) Histomorphology schematic of the hair cycle including anagen, catagen, and telogen stages. Reproduced with permission.^[^ ^195^ ^]^ Copyright 2019, American Chemical Society.

Long et al. developed a self‐activated WIE device for wound healing that significantly accelerated skin wound recovery under the influence of small electric pulses produced by a wearable NG‐based bandage (**Figure** **7**a).^[^ ^190^ ^]^ It consists of two components, including a NG for biomechanical energy conversion and electric pulse generation, and a pair of flexible dressing electrodes. The pair of dressing electrodes was aligned on both sides of the wound, providing an AC electric field that could penetrate into the dermis, and strengthen the endogenous electric field for enhanced wound healing. This self‐powered electric filed could largely accelerate the wound healing rate on rat from 2 weeks to 3 days. It was suggested that the NG‐produced electric fields could facilitate fibroblast cells growth, migration, alignment. It could also improve secretion of three typical growth factors involved in wound healing, including transforming growth factor‐*β*, epidermal growth factor, and vascular endothelial growth factor (VEGF). Besides, the electric pulses from NG produced much lower ROS level compared to normal AC signals, which could be attribute to the charge‐limited voltage generation mechanical of NGs.

Alopecia is another common dermatological disorder due to a growth factor deficiency and/or hair cycle disorder.^[^ ^191^ ^]^ ES was also found having positive effects on alopecia cue to electrotrichogenesis, which could enhance the influx of calcium ions into the dermal papilla cells via voltage‐gated transmembrane ion channels, facilitate ATP synthesis in mitochondria, activate protein kinases, and stimulate protein synthesis and cell division.^[^ ^192^, ^193^, ^194^ ^]^ Based on the proven therapeutic effects of traditional ES on hair growth, Yao et al. fabricated a universal motion‐activated ES device (m‐ESD) that could effectively promote hair regeneration via random body motions.^[^ ^195^ ^]^ Similar to the wound healing bandage, the m‐ESD also consisted of two modules, including an omnidirectional TENG acting as the electric pulse generator and a pair of interdigitated dressing electrode providing spatially distributed electric fields (Figure 7b). The electric field dressing area showed significantly faster hair growth rate as well as higher density of hair follicles compared to hair regeneration medicines (minoxidil and vitamin D). Cell level study revealed that the ES could enhance secretion of VEGF and keratinocyte growth factor (KGF), and thereby alleviate hair keratin disorder, increase the number of hair follicles, and promote hair regeneration on genetically defective nude mice. This work provided a convenient nonpharmacological hair regeneration strategy that may be more effective with fewer side effects for reversing human boldness.

### 4.4 Electroceuticals for Tissue Regeneration

In early 1950s, Fukada and Yasuda demonstrated the piezoelectric effect in bones, where electric potential could be generated when pressure was applied to the bone.^[^ ^196^ ^]^ Based on this discovery, subsequent WIE devices were developed to introduce external electric field to enhance these endogenous electric fields for fracture healing.^[^ ^197^ ^]^ ES has shown its efficacy in a variety of orthopedic conditions such as helping delayed fractures and osteotomies, enhancing the efficacy of bone grafts, treating fresh fractures, and aiding femoral osteonecrosis.^[^ ^198^ ^]^ Three different administrating modes of ES have been practiced, including DC (5–00 µA),^[^ ^197^ ^]^ capacitive coupling (1–10 V, 20–200 kHz, 1–100 mV cm^–1^ electric field),^[^ ^199^ ^]^ and inductive coupling (0.1–20 G, 1–100 mV cm^–1^ pulsed electromagnetic field).^[^ ^200^, ^201^ ^]^ Different mechanisms were proposed to explain the stimulatory effects of these stimulation mode in enhancing fracture healing. DC could lower the oxygen level, generate reactive oxygen species (ROS) and increases the pH, which could accelerate osteoblast cell proliferation.^[^ ^202^, ^203^, ^204^, ^205^ ^]^ Capacitive coupling would raise the amount of cytosolic calcium through voltage gated calcium channels, which in turn enhances activated calmodulin stores. The enhanced cell proliferation would lead to accelerated callus formation and maturation, facilitating bone healing.^[^ ^206^, ^207^, ^208^ ^]^ Inductive coupling signals would directly increase intracellular calcium, leading to enhanced bone healing.^[^ ^209^, ^210^, ^211^, ^212^ ^]^

**Figure 8:**
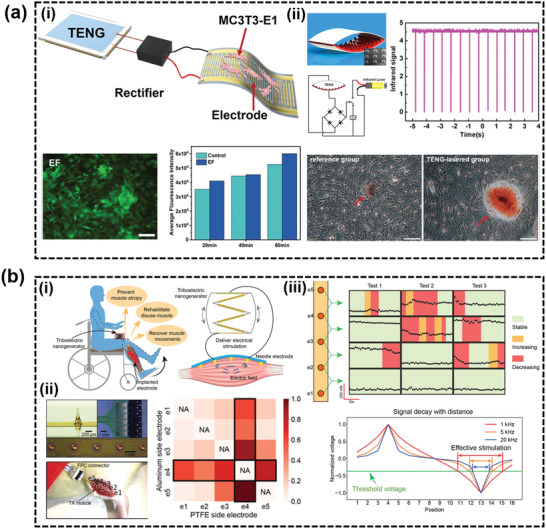
WIEs for bone/muscle stimulation. a) Bone cell stimulation through WIE: i) TENG providing direct stimulation that enhances osteoblasts’ attachment, proliferation, and differentiation by the upregulated level of intracellular Ca^2+^. Reproduced with permission.^[^ ^214^ ^]^ Copyright 2019, Elsevier. ii) WIE stimulating preosteoclasts and promoting its proliferation through an NG‐powered low‐level laser diode. Reproduced with permission.^[^ ^215^ ^]^ Copyright 2015, American Chemical Society. b) Muscle stimulation through WIE: i) Illustration of muscle stimulation directly by TENG‐based WIEs. ii) In vivo stimulation through penetrating electrodes on the rat tibias anterior muscle. iii) Stimulation waveform through TENG‐based WIEs exhibiting stable force profile and recruiting motoneurons located in a larger area around the stimulation electrodes. Reproduced with permission.^[^ ^139^ ^]^ Copyright 2019, Wiley‐VCH.

NGs also showed unique application potential in ES for bone healing (**Figure** **8**a).^[^ ^213^ ^]^ In an in vitro study, Tian et al. demonstrated a significantly promoted osteoblasts attachment, proliferation and differentiation due to the up‐regulated level of intracellular Ca^2+^ under the stimulation of an externally connected TENG.^[^ ^214^ ^]^ Another self‐powered low‐level laser treatment system was also developed to promote osteogenesis.^[^ ^215^ ^]^ The NG‐generated electricity was used to power a low‐level laser diode to stimulate the proliferation of preosteoclasts. A most recent published work revealed that self‐powered WIEs could assist titanium bone implants by enabling anti‐fouling and osteogenesis capability.^[^ ^216^ ^]^ These initial successes suggested that NG could act as a promising self‐powered WIEs for treating bone homeostasis, alleviating osteoporosis and osteoporosis‐related fractures. Since bones are often covered under thick muscle layers, better implantable feature of NGs, particular bioresorbable capabilities are desired for practical applications.

Nerve injuries or neurodegenerative diseases, which are characterized by degeneration of motoneurons, atrophy of skeletal muscles, and generalized weakness, can result in muscle function loss, including muscular atrophy and paralysis.^[^ ^217^ ^]^ ES via waveform generators have been clinically applied to prevent muscle disuse atrophy, and recover muscle movements.^[^ ^218^, ^219^, ^220^ ^]^ As being discussed previously, both PENG and TENG can generate similar electric pulses by harvesting energy from body motions with a typical output voltage of 1–10 V and current of 0.1–1 µA. Therefore, NGs possess a unique potential to function as a wearable or implantable muscle WIE system by simultaneously serving as a waveform generator and power supply.

Wang et al. proposed a self‐powered wearable system of a stacked‐layer TENG and a multiple‐channel epimysial electrode to directly stimulate rat's tibias anterior muscles (Figure 8b).^[^ ^139^ ^]^ The degenerate current waveform of TENGs could effectively avoid synchronous motoneuron recruitment in between the two stimulation electrodes to reduce force fluctuation. It was thus believed that TENG‐driven muscle stimulations could be used for rehabilitative and therapeutic treatments for muscle function loss. Several preliminary demonstrations of TNEG‐enabled nerve/muscle treatments have reported.^[^ ^221^, ^222^, ^223^, ^224^ ^]^ However, the electrode–motoneuron interactions are strongly dependent on the accurate location of the motoneurons. Even identical ES signals and electrode configurations might result in different therapeutic efficacies. Smarter designs on microscale or nanoscale electrodes with accurate mapping and feedback of the motoneurons are highly desired for wearable NG‐driven muscle WIEs.

## 5 Prospects and Conclusion

In conclusion, WIEs are of great importance in modern therapeutics. The NG‐based WIEs stand out with a potential to further improve its flexibility, reliability, and miniaturization. The self‐powering capability brings up novel closed‐loop ES with higher efficiency and lower side effects. This article provides an overview of the most recent development of NG‐based WIEs and their therapeutic applications. Different NGs types and biomimetic design principles based on materials innovation and structure engineering are discussed, together with later broad implementations covering heart, skin, nerve, and bone stimulations, in which treatment mechanisms and posttreatment effects/results are analyzed. While NG‐based WIEs are a booming realm with enormous versatility and possibility, several apparent and daunting challenges need to be addressed for clinical applications.

1. To achieve true self‐powering capability. As discussed in this article, power source remains the key challenge for WIE devices to achieve ultimate minimization and flexibility. NGs are an innovative solution for addressing this challenge. The biomechanical energy could be considered an “infinite” energy source for powering the implanted devices if harvested appropriately and adequately. So far, many improvements have been brought through on the power output of implantable NGs to reach the operational power requirements of implanted devices. Nevertheless, many proof‐of‐concept designs are still far from the practical level in terms of power and energy density, particularly over a long period of time in the biological environment. In order to move one step further toward practical demonstrations, long‐term operation stability is particularly important to show. Specifically, how well can the device sustain under continuous and repeating straining actions when surrounded by a liquid environment without liquid infiltration or electrical leakage? How well can the device maintain a stable and strong interface with the implanted tissue under strong straining conditions? Addressing these critical challenges will lead the promising NG technology to an alternative power source for WIEs.
2. To maintain a long‐term biocompatibility. For an implanted WIE, the long‐term biocompatibility is a critical issue. Although most reported WIEs have evaluated the biocompatibility in vivo in terms of cell cytotoxicity and animal models, few of them reach a living implantation more than a year. It is essential to understand whether the long‐term implantation would have chronic side effects such as inflammation due to long‐time exposure to ES signals, toxicity due to nanoscale or microscale debris from the device, biofouling on the device surface, and interference in normal organ functions. Many new materials, particularly nanoscale materials, have been increasingly developed and used in current WIEs with unprecedented performances. Understanding and improving their long‐term biocompatibility and minimizing the FBR effect become particular significant and urgent. One promising strategy would be surface modification with zwitterionic hydrogels to block protein absorption, the first step of FBR. Besides, to formulate in vivo evaluation criteria of the biocompatibility for the groups of new materials, composites and devices are also needed to point the direction toward clinical applications.
3. To bring new concepts in material and device design to minimize invasivity. Regardless of the flexibility and stretchability that current WIE devices may exhibit, their implantations are mostly done through invasive surgeries. Attachment of large pieces to mobile tissue surfaces may need stiches to stabilize the device to prevent migration under dynamic operation conditions. These invasive operations have been typically used both in laboratory and clinically, yet are not preferred from both physician and patient perspectives. Less‐invasive implantation of miniaturized pacemakers through interventional cardiac catheterization is a good example of signaling the development direction of next‐generation WIEs with easy implantation possibilities. To reach this aggressive target, the size, flexibility, and even foldability need to be more intensively considered in correlation to rather than just the operation conditions but also the implantation conditions. In addition, advanced or smart adhesives are preferred to achieve easy and stable attachment of the implanted device. They might be activated via external optical or acoustic stimulations to convert from non‐sticky transport status to sticky affixing statutes, which can effectively eliminate the invasive suture process.
4. To enable controlled bioresorbability. Many WIEs only require finite operational time, ranging from a few days to months or years, such as bone healing, nerve repair, and drug delivery. They need to be retrieved after the operational lifetime or fulfilling the treatment duty, unfortunately, via an additional surgery. In recent years, bioabsorbable electronics were increasingly developed based on bioabsorbable materials like silk fibroin, cellulose, and chitin. However, functions offered by these groups of materials are rather limited. Materials innovations are needed to introduce more diverse functionalities to the bioabsorbable materials. For example, TENG requires dielectric materials with strong electron interactions. Improving the materials electron affinity and permittivity is a main route to reach high triboelectric output. Piezoelectricity is more naturally associated with biomaterials, such as cellulose, chitin, and amino acids. New strategies to achieve large‐area molecule alignment of this group of material will bring them to the category of high‐performance piezoelectric materials that are feasible for biomechanical energy harvesting and actuation.

In general, the soft and flexible NG‐based WIEs are now showing great promises to revolutionize electroceutical therapeutics through a novel closed‐loop ES manner. By fully addressing the abovementioned challenges, this technology is foreseeable to transform the research and developments in the fields of biomaterials, biomedical engineering, and particularly implantable and wearable medical devices, toward the next generation of precision electrotherapy in the near future.

## Conflict of Interest

The authors declare no conflict of interest.
